# Parental tolerance of risk and associations to adventurous play in British preschool-aged children

**DOI:** 10.1016/j.pmedr.2025.103282

**Published:** 2025-10-14

**Authors:** Kateryna Tyzhuk, Helen F. Dodd, Kathryn R. Hesketh

**Affiliations:** a MRC Epidemiology Unit, Institute of Metabolic Science, Box 285, Addenbrooke's Hospital, Hills Road, Cambridge CB2 0QQ, United Kingdom; b Children and Young People's Mental Health Research Collaboration (ChyMe), Faculty of Health and Life Sciences, University of Exeter, St Luke's Campus, Heavitree Road, Exeter EX1 2LU, UK

**Keywords:** Adventurous play, Risky play, Parents, Preschoolers, Young children

## Abstract

**Objective:**

Adventurous (risky) play may support children's development, but involves injury risk. Parental risk tolerance likely impacts children's play, yet evidence in early childhood is limited. We aimed to examine associations between parental risk tolerance (injury protection, risk engagement, risk tolerance) and adventurous play among British preschoolers.

**Methods:**

Data were from British Preschool Play Survey (February 2023), a nationally representative cross-sectional survey of parents of 2–4-year-olds. Risk tolerance was measured using Risk Engagement and Protection Survey (REPS) and Tolerance of Risk in Play Scale (TRiPS), generating three exposure variables: parental risk engagement (REPS-RE), injury protection (REPS-IP) and risk tolerance (TRiPS). The outcome – hours of adventurous play/week - was derived from Children's Play Scale. Associations were assessed with multivariable linear regressions adjusting for confounders; interactions with child gender were tested.

**Results:**

Caregivers of 1166 children provided valid data. Parental risk engagement (β = 0.68, 95 % CI:0.47,0.89) and risk tolerance (β = 0.07, 95 % CI:0.04,0.10) were positively associated with hours/week of adventurous play. Injury protection was not significantly associated. No interactions were identified.

**Conclusion:**

Supporting parents to navigate child risk is crucial when promoting adventurous play. Interventions are warranted to strengthen parental confidence whilst balancing protection, to allow child exploration and healthy development.

## Highlights

• First study linking parental risk attitudes to adventurous play in preschoolers.
• Parental risk tolerance and engagement are associated with adventurous play.
• These associations are seen for both young boys and girls.
• Parental injury protection is not associated with children's adventurous play.
• Findings can support a balanced approach to risk-benefit assessment in parents.

## 1 Introduction

Play in early childhood has a wide range of benefits. One form, adventurous or risky play, broadly defined as ‘child-led play where children experience subjective feelings of excitement, thrill, and fear’, e.g. climbing trees or rough-and-tumble play (Dodd and Lester, 2021; Sandseter, 2007), is often associated with increased physical activity, better coordination and muscle strength, potentially contributing to improved children's physical health (Brussoni et al., 2015). Adventurous play can also contribute to children's mental well-being through improved self-confidence, resilience, and executive functioning (Sandseter et al., 2023; Hesketh and Dodd, 2023). Engaging in this form of play may be particularly important during early childhood, a time of rapid physical, cognitive, and socio-emotional growth, during which behaviours and habit formation have long-lasting impacts on children's health(National Health Service (NHS) Digital, 2023; Pitchforth et al., 2019).

Yet despite emerging evidence on the benefits of adventurous play for children's health, opportunities for risk and adventure in play have decreased over the last two decades. This decline has been reported in the USA (Kybartas et al., 2024), New Zealand (Jelleyman et al., 2019), and even Norway (Sandseter and Sando, 2016), known for its high risk tolerance in children's play. A 2022 British survey indicated that only 27 % of children engage in outdoor play that potentially involves risky and adventurous activities, compared to 71 % of the “baby boomer” generation (Save the Children UK, 2022). This decline has led the UN Committee on the Rights of the Child to raise concerns about poor recognition of its General Comment No. 17, Article 31 (UN Committee on the Rights of the Child, 2013), which addresses a child's fundamental right to play, as governments often prioritise safety regulations and academic performance instead.

In addition to the societal-level influences on children's play, the family is likely to have a strong impact, particularly in young children. Parental influence on children's play may be more significant now than in the past, as the amount of time British parents spend with their children has increased by 250 %, from an average of 23 min per day in 1975 to 80 min in 2015 (Richards et al., 2016). Low parental risk tolerance, often shaped by social pressures and fear of injuries (Oliver et al., 2023), may lead parents to prioritise protection over their child's natural seeking of exploration and adventure. As a result, limited opportunities for risky and adventurous activities may have long-term detrimental effects on preschoolers. Boys, who are more inclined toward adventurous forms of play, including rough-and-tumble play, may be particularly affected by these restrictions (Haidt, 2024). Conversely, evidence shows that preschool-aged girls engage in less adventurous play than boys and are less likely to play outdoors (Morrongiello and Hogg, 2004). Together, this highlights the importance of considering child's gender in research on adventurous play. Parental risk tolerance may also be influenced by a child's gender with parents often discouraging risk-taking in girls compared with boys, as seen in school-aged children (Olsen et al., 2019; Sandseter et al., 2021). Parents may also provide “safer” activities, which are more sedentary and screen-based (Bundy et al., 2011), offering less physical activity and social engagement. With the decline of adventurous play and the increase in time that parents and children spend together, there is a growing need to understand how parental risk tolerance affects children's opportunities for adventurous play and whether child gender determines this relationship.

To date, research examining the association between parental risk tolerance and children's adventurous play has mostly focused on older children. In a representative sample of British 5–11-year-old children a positive association between parental risk engagement and tolerance, and children's adventurous play was noted, although no association was found for parental injury protection (Dodd et al., 2021a). These results were partially replicated in an Australian sample of parents of 5–12-year-olds (Jerebine et al., 2024), although parental risk tolerance, engagement and injury protection were all associated with the children's adventurous play. Importantly, no study of younger children has specifically examined how parental risk tolerance is associated with children's adventurous play. This is important because what constitutes a risk in young children may be perceived differently, and parents are likely to be the primary ‘gatekeepers’ for adventurous play (Oliver et al., 2023).

This study, therefore, aimed to explore the relationship between parental risk tolerance (i.e., injury protection, risk engagement and risk tolerance) and time spent playing adventurously in British preschool-aged children. We hypothesised that children whose parents were more risk-tolerant and encouraged risk engagement would have higher levels of adventurous play, while parental injury protection would be negatively associated with adventurous play. We also assessed whether parental risk tolerance interacted with child gender, hypothesising that the relationship between tolerance and adventurous play would be stronger for girls.

## 2 Methods

### 2.1 Study design and procedure

Data were from the British Preschool Play Survey (BPPS) (Dodd and Hesketh, 2024), a representative cross-sectional online survey assessing play behaviours of British preschool-aged children aged 2–4. The preschool survey is a modified version of the British Children's Play Survey focused on children aged 5–11. Data were collected in February 2023 by YouGov UK, a public opinion research company with over one million panellists. Survey invitations were emailed to YouGov UK panel members who met the inclusion criteria of being parents of children aged 2–4 years. The final dataset comprised responses of 1166 parents and was weighted to the national population according to age, gender, education, social grade, residency region and location, as provided by YouGov UK. Participants were requested to report on activities of their eldest child within the investigated age range over the past year. Ethical approval was provided by the University of Cambridge (HSSREC.22.312). Informed consent was obtained from all participating parents prior to survey completion. The descriptive findings from the BPPS have been published elsewhere (Dodd and Hesketh, 2024).

### 2.2 Measures

#### 2.2.1 Outcome variable

The Children's Play Scale (CPS) was used to generate the outcome variable: total hours/week of adventurous play. The CPS (Dodd et al., 2021b) measures children's play across seven locations – home, outside home (e.g. garden), playground, green spaces (e.g. park), street or public space, indoor play centre (e.g. trampoline park) and outdoors near open water (e.g. lake). Parents retrospectively report on their child's play in each location during the Autumn-Winter and Spring-Summer seasons, using a 7-point Likert scale for play frequency (from ‘every day’ to ‘never’) and a 6-point scale for duration (from ‘less than 30 min’ to ‘more than 4 h’). Additionally, they report the adventure level at each location using a 5-point scale, from ‘very low levels of adventure’ to ‘maximum adventure’. The scoring method from a previous study on older children (Dodd et al., 2021a) was applied to estimate the total hours of adventurous play per year. First, the play frequency variable was transformed, e.g., ‘every day’ became 182.5 days (365/2), and ‘4–6 times/week’ became 130 days (26 weeks*5 times). The duration scores were similarly converted, e.g., ‘half an hour to 1 hour’ became 0.75 h. The play frequency and duration variables in each place were then multiplied, generating the total hours spent playing in each location for Autumn/Winter and Spring/Summer seasons. Adding these two numbers together produced an estimation of the total hours spent playing per year in each place. The outcome variable, total hours/week of adventurous play, was then calculated using only those places that parents rated at least a ‘mild level of adventure’ (score two on a 5-point scale), a threshold previously used in older children (Dodd et al., 2021a). This was then divided by 52 (weeks in a year) to aid interpretation.

#### 2.2.2 Exposure variables

We used the following two scales to derive three exposure variables.

Risk engagement and protection survey (REPS)

The REPS (Olsen et al., 2019) is a self-report measure evaluating parental attitudes toward risk engagement and injury protection in children's play. It consists of 14 statements rated on a 7-Likert scale from 'very strongly disagree' (Dodd and Lester, 2021) to 'very strongly agree' (Pitchforth et al., 2019). Following Jelleyman et al. (Jelleyman et al., 2019), scores were calculated using 12 out of 14 initially suggested items: six for risk engagement (REPS-RE, e.g. *It is important for my child to engage in physically challenging experiences*) and six for injury protection (REPS-IP, e.g. *I am concerned about the potential hazards in my home*). Each subscale ranges from 6 to 42, with higher scores indicating greater risk engagement and injury protection. The risk engagement subscale had α of 0.9, and the injury protection - 0.78, indicating high internal consistency.

Tolerance of risk in play scale (TRiPS)

The TRiPS (Hill and Bundy, 2014) measures parents' risk tolerance in children's play using 31-item questionnaire based on Sandseter's risky play categories (Sandseter, 2007). The scale was validated in parents of children aged 3–13 and contains questions about risky scenarios, e.g. *Would you let the child climb up a tree within your reach?* The scores range from 0 (‘no’) to 1–12 (‘yes’) based on risk acceptability, i.e., less acceptable items scoring higher than more acceptable determined by a Rasch analysis (Hill and Bundy, 2014). These responses are summed to generate a parental risk tolerance score, ranging from 0 (least risk-tolerant) to 184 (most risk-tolerant).

#### 2.2.3 Additional covariates

Additional covariates were identified through a comprehensive literature review, their availability in the dataset and previously reported association with adventurous play. Directed Acyclic Graphs (DAGs) were generated using DAGitty (Textor et al., 2016) to guide covariate inclusion in the models, ensuring a theoretically grounded approach (see Supplementary material A). Parental covariates included age (<35 years vs ≥35 years), social class (middle class vs working class), education level (low/none, medium, high), ethnicity (White British vs minority ethnic group), disability status (yes/no), residency location (urban, town, rural), and region (England North, Midlands, East, South, London, Wales, Scotland). Child covariates included gender (male/female), age (2, 3 or 4 years), birth order (first, second, third or later), learning disability (yes/no), and physical development (less capable, same as peers, more capable). Hours in childcare was included as a continuous variable. Categories with low numbers of respondents were collapsed (see Results).

### 2.3 Statistical analysis

The dataset is available via https://doi.org/10.5255/UKDA-SN-9308-1. Variable distributions were assessed before analyses. To mitigate the effect of outliers on the outcome, total hours/week of adventurous play, the data were winsorized between the 5th and 95th percentiles (Jerebine et al., 2024; Dodd and Hesketh, 2024).

Three multivariable linear regression models were used to explore the association between each exposure variable (parental risk tolerance) and total hours/week of adventurous play. Each model was fitted using the *survey* package v.4.2–1 (Lumley, 2004) to account for weighted survey data. Standard linear model assumptions were tested and found to be appropriate. Collinearity between the three exposure variables was not present (Pearson correlation coefficients all below *r* < 0.7). All models included relevant covariates as per DAGs - parent age, social class, education, ethnicity, disability, residency location and region, as well as child gender, age, birth order, learning disability, physical development, and hours in childcare. Interaction terms were included in each model to assess differences in associations based on children's gender (Morrongiello and Hogg, 2004; Olsen et al., 2019) and retained if significant. We conducted a post hoc sensitivity analysis to determine whether findings differed when using total hours/week of adventurous play calculated using places parents rated as moderately adventurous (3/+ out of 5) instead of mild (2/+ out of 5, as per main analyses). Details on missing data are provided in Supplementary material B. All analyses were conducted in March–June 2023 in R 4.4.0 using R Studio 023.04.1 + 748 (RStudio Team, 2022).

## 3 Results

A total of 1166 parents (66 % mothers) with children aged 2–4 years (M_age_ = 3.06, SD = 0.8; 52 % boys) provided valid data. Most parents were ≥ 35 years (65 %), middle-class (63 %), and with high levels of education (55 %). The sample was predominantly White British (88 %), residing in England (87 %) and urban-dwelling (79 %). Just under 20 % of parents reported having a disability, 7 % indicated their child's learning disability and 10 % child having lower physical development. Table 1 shows the complete demographic profile of the surveyed population.

**Table 1:** Demographic characteristics of the participating parents, British Preschool Play Survey, February 2023.

DEMOGRAPHICS	n	%
**Child**		
Child gender		
Male	604	52
Female	562	48
Child age (*N* = 1166)		
2 years old	327	28
3 years old	418	36
4 years old	421	36
Child birth order (*N* = 1166)		
First-born	632	54
Second-born	379	33
Third-born or later	155	13
Child learning disability (*N* = 1072)⁎		
No	1000	93
Yes	72	7
Child physical development (*N* = 1166)		
More capable than peers	478	41
Same as their peers	571	49
Less capable than peers	117	10
		
**Parent**		
Parent age (*N* = 1166)		
Under 34	413	35
35 and older	753	65
Social class (*N* = 1166)⁎⁎		
Middle class (ABC1)	739	63
Working class (C2DE)	427	37
Education level (*N* = 1137)⁎		
Low or no formal education	176	15
Medium	340	30
High	621	55
Parent ethnicity (*N* = 1144)⁎		
White British	1003	88
Minority	141	12
Region (*N* = 1166)		
England	1010	87
*London*	*273*	*27*
*England North*	*203*	*20*
*England Midlands*	*112*	*11*
*England East*	*137*	*14*
*England South*	*285*	*28*
Wales	54	5
Scotland	102	9
Location (*N* = 1160)⁎		
Urban	919	79
Town or Fringe	128	11
Rural	113	10
Parent disability (*N* = 1140)⁎		
No	936	82
Yes	204	18

⁎ Some participants either indicated they were unsure (marked as “don't know”) or chose not to disclose this information (marked as “prefer not to say”). This was marked as missing.

⁎⁎ The social grade of the respondents was determined as a middle class (ABC1) or working class (C2DE) as classified by YouGov UK.

Summary statistics for parental risk tolerance exposure variables, i.e. injury protection, risk engagement and risk tolerance, and weekly adventurous play (outcome variable) are in Table 2. The mean scores were 26.7 (0.16) for REPS-IP, 30.5 (0.17) for REPS-RE, and 63.2 (1.01) for TRiPS. Additionally, preschool-aged children spent an average of 22.4 (0.51) hours per week playing adventurously.

**Table 2:** Summary statistics for parental risk tolerance measures (exposure variables) and adventurous play of preschool-aged children (outcome variable), British Preschool Play Survey, February 2023.

Variables	Mean (SE)
Injury protection REPS-IP⁎ (range 6–42)	26.7 (0.16)
Risk engagement REPS-RE⁎ (range 6–42)	30.5 (0.17)
Risk tolerance TRiPS⁎ (range 0–184)	63.2 (1.01)
Average weekly adventurous play (hours)	22.4 (0.51)

⁎ REPS-IP = Risk Engagement and Protection Survey – Injury Protection subscale; REPS-RE = Risk Engagement and Protection Survey – Risk Engagement subscale; TRiPS = Tolerance of Risk in Play Scale.

Regression analyses (see Table 3) revealed positive associations between parental risk tolerance (REPS-RE and TRiPS) and preschoolers' adventurous play. REPS-RE was positively associated with total hours of adventurous play per week (β = 0.68; CI: 0.47,0.89), indicating that for every one-point increase in parental REPS-RE score, the duration of their children's adventurous play increased by an average of 0.68 h, or almost 40 min per week. Similarly, TRiPS was also positively associated (β = 0.07, 95 % CI: 0.04,0.10), such that each one-point increase in TRiPS score (range of 0–184) was associated with an increase of 0.07 h, or around 4 min, in adventurous play per week. REPS-IP was not significantly associated with the total hours/week of adventurous play. No significant interaction effects between children's gender and variables of interest were identified (see Supplementary material C). Sensitivity analyses indicated that findings were very similar when analyses were conducted using at least moderate (vs. at least mild) levels of adventurous play as an outcome, with REPS-IP being negatively associated with adventurous play (see Supplement material D).

**Table 3:** Results for three multivariable linear regressions examining the association between parental risk tolerance and the total hours/week of adventurous play among preschoolers, British Preschool Play Survey, February 2023.

Exposures	Unadjusted β (95 % CI)			Adjusted β (95 % CI)*		
(Intercept)	27.36 (21.85, 32.87)	2.52 (−2.76, 7.80)	17.48 (15.30, 19.66)	32.90 (25.77, 40.04)	7.48 (0.10, 14.87)	23.67 (18.82, 28.53)
REPS-IP°	−0.18 (−0.39, 0.03)	–	–	−0.18 (−0.39, 0.02)	–	–
REPS-RE°	–	0.65 (0.48, 0.83)	–	–	0.68 (0.47, 0.89)	–
TRiPS°	–	–	0.08 (0.05, 0.11)	–	–	0.07 (0.04, 0.10)

*Analyses adjusted for children's gender, age, birth order, learning disability, physical development, hours in childcare and parents' age, social grade, education, ethnicity, disability, region and location of living

° REPS-IP = Risk Engagement and Protection Survey – Injury Protection subscale; REPS-RE = Risk Engagement and Protection Survey – Risk Engagement subscale; TRiPS = Tolerance of Risk in Play Scale.

## 4 Discussion

This is the first study to quantitatively investigate the association between parental risk tolerance and total hours/week of adventurous play in preschool-aged children. Regression analyses revealed that two of three measures of parental risk tolerance – REPS-RE and TRiPS - were positively associated with preschoolers' adventurous play, indicating that higher parental risk engagement and tolerance are associated with higher levels of adventurous play among young children. Considering the positive effects of adventurous play on children's physical and mental health (Dodd and Lester, 2021; Brussoni et al., 2015; Sandseter et al., 2023; Hesketh and Dodd, 2023), this highlights that parents may play a crucial role in providing opportunities for adventurous play, contributing to their children's healthy development.

Aligned with these results, previous research in older children (Dodd et al., 2021a; Jerebine et al., 2024) identified a similar relationship between parental risk tolerance and adventurous play, such that REPS-RE and TRiPS were positively associated with adventurous play among school-aged children. This is perhaps unsurprising given the amount of time that (young) children spend with parents and that parental influence is one of the main determinants of children's decision-making in their adventurous or risky play (Morrongiello and Lasenby-Lessard, 2007). This is despite evidence suggesting that parents often find themselves dealing with conflicting feelings related to adventurous play. On one hand, they recognise potential benefits, highly value it as a tool to develop children's independence and competence, and, therefore, tolerate certain risks in their children's play (Waddington and Pearson, 2022). However, bound by a legal duty of care, it is also their responsibility to ensure their children's safety and well-being, particularly at younger ages, when children are still developing essential life skills, which may limit parental desire to permit adventurous play. This can be compounded by other barriers, such as social pressures and the fear of being perceived as “bad parents”, collectively resulting in lower risk tolerance, increased supervision and consequently, perhaps less adventurous play for their children (Visser et al., 2024).

Despite these concerns, our findings also indicate that parental injury protection in children's play (REPS-IP) was not significantly associated with the amount of adventurous play among 2–4-year-olds. This suggests that while parents may struggle to balance children's opportunities for adventure or risk in play with ensuring children's safety (Niehues et al., 2015), their approach to injury protection does not necessarily translate into restrictions on adventurous play. This study, therefore, adds to the growing body of research that emphasises the complex nature of parental risk tolerance in children's play. It also provides evidence that while higher parental tolerance is potentially related to greater opportunities for adventurous play, even in the youngest age groups of children, parental injury protection does not appear to restrict it.

Contrary to previous research in older children, gender did not moderate the association between parental risk tolerance and adventurous play. This is despite girls tending to engage in less adventurous play than boys in this preschool cohort (Dodd and Hesketh, 2024) and older age groups (Dodd et al., 2021a). Parents of younger children may be equally conscious about the safety of their sons and daughters, and more likely to be influenced by their child's developmental milestones and individual risk disposition rather than their gender, as previously shown by qualitative research (Creighton et al., 2017). Gender differences in risk tolerance may emerge later as children gain more independence in their play. Future research aimed at understanding how child age and gender may be associated with parental risk tolerance will help understand how to promote adventurous play in both boys and girls.

Taken together, this work contributes to the increasing evidence about how parents can shape children's opportunities for adventurous play. Further research is needed to understand why some parents are more risk-averse than others, particularly among the youngest age group. Doing so will provide evidence on how best to support parents and find a balance between their duty of care and the desire to allow their children to engage in adventurous play. For instance, parents could practice pausing for a moment to observe children's “state of play” before intervening, focusing on allowing their child to build awareness of the situation and manage risks (Play Safety Forum, 2012). In addition, helping parents overcome potential barriers to adventurous play, e.g. social pressure, safety, infrastructure accessibility (Oliver et al., 2023), and recognising the difference between fostering a child's exploration under guidance and exposing children to risks without proper supervision will likely support children's adventurous play. Initial attempts to address these objectives in older children, such as the Sydney Playground Project (SPP) in Australia (Bundy et al., 2011) and “Go Play Outside!” in Canada (Brussoni et al., 2021), have proven effective. This also aligns with broader recommendations, such as those from the UK National Institute for Health and Clinical Excellence, advocating for children “to develop skills to assess and manage risks, according to their age and ability” (National Institute for Health and Care Excellence (NICE), 2013), as well as the UK Play Safety Forum (Play Safety Forum, 2012), which emphasises the importance of keeping children as safe as necessary and not as safe as possible. Further research is now required to understand whether similar initiatives could be effective in the preschool population. By intervening earlier, it may be possible to provide better support for children from earlier ages, helping them grow into more resilient young adults.

### 4.1 Limitations and strengths

Several limitations of this study should be acknowledged. The cross-sectional design limits causal inferences, and potential residual confounding (e.g., variation in parental risk perceptions) may remain despite multiple covariate adjustments. Parent-reported data may introduce recall and social desirability biases, and parents may perceive activities to have differing levels of risk based on their own perceptions. Whilst this should be borne in mind when interpreting findings, sensitivity analyses suggested that coefficients and findings were similar when using at least moderately (vs. at least mildly) adventurous locations, suggesting our findings are robust regardless of adventure level. Missingness in the outcome variable was non-random, mainly from parents selecting “don't know” responses, with fewer usable data from lower socio-economic and minority ethnic families. Using sample weights, correcting to the UK's population average, minimised sampling bias. Finally, generalisability may be limited as neither REPS nor CPS has established test-retest reliability or construct validity in parents of preschoolers.

Nevertheless, this is the first quantitative study to investigate associations between parental risk tolerance and total hours/week of adventurous play in preschoolers, using validated measures. Use of a large, nationally representative sample allows comparison and provides important information about how parental perceptions are associated with children's play behaviours.

## 5 Conclusion

This study found that higher parental risk engagement and tolerance are associated with higher levels of adventurous play among young children, highlighting the important role that parents may have in enabling adventurous play in young children. As children's opportunities for adventurous play seem to be declining, this work provides valuable context to inform the development of targeted interventions intended to balance the necessity of safety with the benefits of allowing children to take risks under proper supervision. By empowering parents with the knowledge and tools that facilitate safe yet adventurous play, we can promote healthier children who are better equipped to assess and manage risks throughout their lives.

## CRediT authorship contribution statement

**Kateryna Tyzhuk:** Writing – original draft, Methodology, Formal analysis, Data curation, Conceptualization. **Helen F. Dodd:** Writing – review & editing, Supervision, Methodology, Data curation, Conceptualization. **Kathryn R. Hesketh:** Writing – review & editing, Supervision, Project administration, Methodology, Formal analysis, Data curation, Conceptualization.

## Funding

This work was supported by the 10.13039/501100000269UKRI Economic and Social Research Council (grant number ES/P000738/1); the UKRI Future Leaders Fellowship (grant numbers MR/Y019164/1, MR/X015033/1, MR/S017909/1) and the 10.13039/501100000265Medical Research Council [grant number MC_UU_00006/5].

## Declaration of competing interest

The authors declare the following financial interests/personal relationships which may be considered as potential competing interests: Kateryna Tyzhuk reports financial support was provided by UKRI Economic and Social Research Council. Dr. Kathryn Hesketh reports financial support was provided by UKRI Future Leaders Fellowship. Dr. Kathryn Hesketh reports financial support was provided by UK Research and Innovation Medical Research Council. Dr. Kathryn Hesketh reports financial support was provided by Wellcome Trust. Dr. Helen Dodd reports financial support was provided by UKRI Future Leaders Fellowship. Dr. Helen Dodd reports a relationship with Play England that includes: non-financial support.

## Data Availability

Data will be made available on request.
